# Relative Validity of Food Frequency Questionnaires in Adult Populations: A Systematic Review and Meta‐Analysis

**DOI:** 10.1002/fsn3.72381

**Published:** 2026-09-30

**Authors:** Carmelo Pujia, Yvelise Ferro, Alessia Placanica, Rossella Bruno, Fiorella Guadagni, Giulia Malaguarnera, Elisa Mazza, Tiziana Montalcini, Arturo Pujia

**Affiliations:** ^1^ O.U. Clinical Nutrition Renato Dulbecco Hospital Catanzaro Italy; ^2^ Department of Medical and Surgical Science University Magna Grecia Catanzaro Italy; ^3^ Department of Clinical and Experimental Medicine University Magna Grecia Catanzaro Italy; ^4^ Department of Human Sciences and Quality of Life Promotion San Raffaele Roma Open University Rome Italy; ^5^ Research Center for the Prevention and Treatment of Metabolic Diseases University Magna Grecia Catanzaro Italy

**Keywords:** 24‐h recall, food frequency questionnaire, food record, meta‐analysis, validity

## Abstract

Accurate dietary assessment is essential in nutritional research and epidemiology. Food Frequency Questionnaires (FFQs) are widely used to estimate habitual intake; however, their performance may vary according to questionnaire design, administration procedures, and the dietary components being assessed. This systematic review and meta‐analysis evaluated the relative validity of FFQs designed to assess overall dietary intake in adults and explored methodological characteristics associated with their performance. A meta‐analysis was conducted on studies validating FFQs against reference methods, including 24‐h recalls and food records. A comprehensive search of the PubMed, Web of Science, and Scopus databases was conducted for publications from January 2000 to February 1, 2026. Pooled estimates were calculated for correlation coefficients and mean differences for energy and nutrients between FFQs and reference methods. The literature search identified 43 articles that included 8467 participants. Subgroup analyses according to the sample size, administration methods, FFQ items, quality of the studies, and regions were also performed. Overall, FFQs showed moderate‐to‐good relative validity for most nutrients. Performance was relatively consistent for energy and macronutrients, particularly carbohydrates, whereas greater variability was observed for micronutrients, although some, including magnesium and riboflavin, showed high correlations. Systematic overestimation of certain nutrient intakes was observed, particularly when FFQs were compared with 24HRs. Interviewer‐administered FFQs and questionnaires with a greater number of items tended to yield slightly higher correlations, while studies of higher methodological quality also tended to report stronger correlations. These findings support the use of FFQs primarily for ranking individuals according to habitual dietary intake and suggest that administration mode, questionnaire comprehensiveness, and methodological quality should be carefully considered in future FFQ development and validation. Greater caution may be warranted when overall‐diet FFQs are used to estimate specific micronutrient intakes.

## Graphical abstract

This meta‐analysis demonstrates that digital Food Frequency Questionnaires are reliable tools for ranking habitual dietary intake in adults. Validation against 24‐h recalls and food records shows moderate‐to‐good correlation coefficients, particularly for carbohydrates and fiber. Factors such as interviewer administration and higher item counts enhance tool performance.

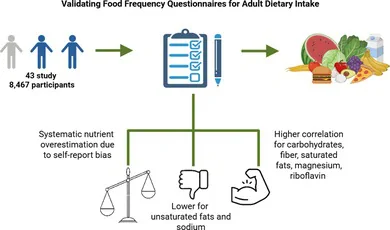

## 1 Introduction

Dietary intake assessment represents a fundamental tool in nutritional research, clinical dietetics, and epidemiology. Diet is a key determinant of health and the risk of non‐communicable chronic diseases such as obesity, Type 2 diabetes, cardiovascular diseases, cancer, and kidney disorders. Therefore, the ability to measure dietary intake accurately is essential to understand the relationships between diet and health, guide nutritional interventions, and inform effective public health policies (Aea et al. 2019; Willett 2013).

Dietary intake can be measured using various methodologies, generally classified into prospective (or real‐time) methods and retrospective methods (Thompson and Subar 2017; Willett 2013). Prospective methods, such as the food diary (or food record), require participants to record every food and beverage at the time of consumption (Thompson and Subar 2017; Willett 2013). This approach has the advantage of minimizing memory‐related errors and, in the weighed diary version, is considered one of the most accurate methods for estimating current energy and nutrient intake (Cade et al. 2002; Thompson and Subar 2017). However, the high participant burden and the need for specific training may lead to unintentional alterations in habitual dietary behavior during the recording period (Cade et al. 2002; Willett 2013).

Retrospective methods, on the other hand, rely on the participant's ability to recall past intake (Cade et al. 2002; Willett 2013).

Among these, the 24‐h dietary recall involves a detailed description of the previous day's food intake through a structured interview (Abutbul Vered et al. 2022; Thompson and Subar 2017). Due to high intra‐individual variability, multiple recalls are often required to accurately estimate an individual's habitual intake (Kipnis et al. 2003; Thompson and Subar 2017).

In this context, the Food Frequency Questionnaire (FFQ) stands out for its ability to assess long‐term intake (weeks, months, or years), making it the instrument of choice for epidemiological studies investigating the relationship between diet and chronic diseases (Cade et al. 2002; Willett et al. 1985). Finally, to overcome the intrinsic limitations of self‐reported methods, such as memory bias or the tendency to underreport, nutritional research often relies on nutritional biomarkers, which serve as objective reference measures for the validation of dietary assessment tools (Kipnis et al. 2003). Among the most commonly used methods to estimate usual dietary intake, the Food Frequency Questionnaire (FFQ) plays a central role due to its ability to capture habitual consumption of foods and nutrients over medium‐ to long‐term periods, its low time requirements, cost‐effectiveness, and ease of use in large‐scale epidemiological studies (Cade et al. 2002; Willett et al. 1985). FFQs are subject to significant measurement errors, related to participants' difficulties in recalling and averaging dietary intake over extended periods, as well as systematic biases that can lead to attenuation of risk estimates and reduced statistical power (Freedman et al. 2011). They generally consist of a number of items, typically ranging from 100 to 180, representing the most frequently consumed foods in the population, with each food item assessed for both consumption frequency and approximate quantity. In recent years, the increasing digitization of nutritional research has led to the development of dietary assessment tools in digital format, administered via web applications or computerized platforms.

These tools have the potential to facilitate standardized data collection, reduce participant burden, integrate digital features such as images for portion size estimation and automated nutrient calculations, and may improve acceptability among participants familiar with digital technologies (Carter et al. 2015; Illner et al. 2012).

Validation is essential because errors in dietary measurement can weaken or obscure the associations between diet and health. Classic studies have shown that both systematic and random errors in FFQs can reduce statistical power and introduce significant bias in epidemiological findings (Kipnis et al. 2003).

Given the established role of FFQs in nutritional epidemiology, the key methodological question is not whether these instruments are useful, but rather which characteristics contribute to better performance and for which dietary assessment purposes they are most appropriate. This issue is particularly relevant for FFQs designed to assess the overall diet, as their ability to estimate nutrient intake may differ between broad dietary components, such as energy and macronutrients, and individual micronutrients. Therefore, the present systematic review and meta‐analysis aimed to evaluate the relative validity of overall‐diet FFQs against established reference methods and, importantly, to identify methodological and design characteristics associated with FFQ performance. Specifically, pooled correlation coefficients and standardized mean differences were evaluated, while subgroup analyses explored the influence of sample size, administration mode, questionnaire length, methodological quality, and geographical setting. These analyses aimed to identify factors associated with stronger relative validity and provide practical guidance for the use of FFQs in nutritional epidemiology.

## 2 Materials and Methods

### 2.1 Selection and Search Strategy

This meta‐analysis was conducted following the guidelines of the PRISMA (Preferred Reporting Items for Systematic Reviews and Meta‐Analyses) (Page et al. 2021). Studies were identified through systematic searches of the following electronic databases: PubMed, Web of Science, and Scopus. The search included articles published between January 2000 and February 1, 2026. The complete database‐specific search strings, including Boolean operators and filters applied, are reported in Table S1. Briefly, the search combined terms for “FFQ”/“Food frequency questionnaire” with “validation”/“validity” in the title/abstract or topic fields, restricted to the January 2000–February 2026 window and to publications in English, with no additional database‐level filters applied.

### 2.2 Inclusion and Exclusion Criteria

This meta‐analysis was designed to evaluate the validity of FFQs developed for epidemiological purposes. In particular, the study focused on FFQs used to assess dietary intake and validated against other dietary assessment methods, such as 24HR and FR. The inclusion criteria were defined as follows. Eligible studies were those describing dietary assessment methods developed for epidemiological purposes and validating FFQs against other dietary assessment methods, such as 24HR or FR. Studies were required to aim at measuring nutrient intake and to be conducted on healthy adult populations aged 18 years or older. In addition, only studies in which the validation of FFQs was assessed using Pearson or Spearman correlation coefficients were considered eligible. Furthermore, studies had to be published in English and conducted in populations from Europe, the United States, Canada, New Zealand, or Australia. If it was not clear from the abstract whether an article met the inclusion criteria, the full text was retrieved and examined. These geographical restrictions were applied to reduce methodological heterogeneity by including studies conducted in settings with broadly comparable dietary assessment practices, food composition databases, and FFQ development frameworks. This approach was intended to improve the comparability of validation results across studies rather than to imply uniformity of dietary habits across all included regions. Specifically, the regions included (Europe, United States, Canada, Australia, New Zealand) share comparable infrastructure for dietary assessment research, namely (i) mature, harmonized national/regional food composition databases (e.g., the USDA National Nutrient Database, the UK McCance and Widdowson's tables, EuroFIR‐harmonized databases in Europe, the Australia/New Zealand FSANZ database, and the Canadian Nutrient File), and (ii) a long‐standing tradition of FFQ development and validation frameworks (e.g., the Willett‐type and Block‐type FFQs and their regional adaptations) following broadly comparable methodological conventions. This comparability of measurement infrastructure, rather than an assumption of dietary homogeneity across these regions, was the basis for the geographic restriction, so as to limit an additional source of methodological (rather than purely dietary) heterogeneity in the pooled estimates.

The exclusion criteria for the meta‐analysis included studies focusing on participants with diseases or specific population groups, such as pregnant women or patients with metabolic syndrome, as well as studies examining nutrient–disease relationships. Studies using FFQs specifically designed to assess individual nutrients (e.g., folate, vitamins, calcium, fats, or proteins) or single food items were also excluded. Additionally, studies recruiting fewer than 50 participants were not considered. Finally, studies comparing FFQs with other FFQs or with biomarkers were excluded from the analysis.

All retrieved studies were examined, and the titles and abstracts were independently screened by two researchers (Y.F. and C.P.) using the Rayyan platform in order to identify duplicate records and evaluate their eligibility according to the predefined inclusion criteria. Articles considered unsuitable were further assessed by a third researcher (A.P.) to verify the appropriateness of their exclusion. In cases of disagreement between the two reviewers, the final decision was made after consultation with the third researcher. Subsequently, the full texts of all potentially eligible studies were independently reviewed by the same authors and discussed to reach a final consensus.

### 2.3 Data Extraction and Quality Assessment

Data were selected and extracted independently by two experienced reviewers (A.P. and C.P.). The following information was collected from each study: authors, title, year of publication, country, population characteristics (including sample size, age, and sex distribution), characteristics of the FFQ, such as the number of food items, the reference period, and the mode of administration, as well as the characteristics of the reference methods used (24HR or FR). In addition, the statistical methods employed to evaluate the validity between the FFQ and the reference methods were recorded.

Data related to the correlation and agreement between the FFQ and the reference methods were considered. In particular, the mean and standard deviation (SD), as well as Pearson or Spearman correlation coefficients (including crude, energy‐adjusted, and de‐attenuated coefficients), were extracted whenever available.

Values were extracted for energy and for the following nutrients: carbohydrates, proteins, lipids, fiber, saturated fatty acids (SFA), monounsaturated fatty acids (MUFA), and polyunsaturated fatty acids (PUFA). Information was also collected for vitamins, including vitamin A, retinol, vitamin C, vitamin D, vitamin E, vitamin K, thiamine, riboflavin, niacin, pantothenic acid, vitamin B6, folate, and vitamin B12, as well as for minerals such as selenium (Se), magnesium (Mg), calcium (Ca), iron (Fe), iodine (I), zinc (Zn), copper (Cu), potassium (K), phosphorus (P), sodium (Na), and manganese (Mn). However, with regard to differences in means, data were not available for iodine, zinc, copper, phosphorus, and manganese. The methodological quality of the validation studies was assessed using the scoring system proposed by Serra‐Majem et al. (2009), which evaluates several methodological aspects, including the characteristics and size of the study sample, the statistical methods used to assess validity, the procedures adopted for data collection, and the consideration of seasonal variation in dietary intake. Based on the total score obtained, studies were classified into four quality categories: very good (≥ 5.0), good (3.5 ≤ score < 5.0), acceptable (2.5 ≤ score < 3.5), and poor (< 2.5).

### 2.4 Statistical Analysis

The meta‐analysis included all studies reporting relevant outcomes and incorporated data on the endpoints considered in the present study. Spearman correlation coefficients do not follow a normal distribution; therefore, they were transformed using the Fisher *r*‐to‐*z* transformation in order to obtain *z*‐values that are approximately normally distributed. The transformation of the correlation coefficient (*r*) to Fisher's *z* was performed according to the following formula: *z* = 0.5 × ln[(1 + *r*)/(1 − *r*)]. The standard error of *z* was then calculated using the following formula: SE = 1/√(*n* − 3) (Muehlbauer et al. 2015). After applying Fisher's *z* transformation, a meta‐analysis was conducted to combine data from the different studies (Wilson and Lipsey 2001). Pooled correlation coefficients and their corresponding 95% confidence intervals (CI) were then calculated. When a study reported separate values for men and women, these were treated as two independent observations within the analysis, taking into account the sample size of each group. The standardized mean differences (SMDs) were calculated by subtracting the nutrient intake levels measured using the reference methods (24HR or FR) from those obtained through the FFQ. For means and SD, SMDs and their corresponding 95% CI were used to represent the effect size of the quantitative data. When the mean and standard deviation were not available in some studies, these parameters were estimated from the median and interquartile range, according to the method proposed by Wan et al. (2014). Furthermore, the heterogeneity among studies was assessed by calculating the inconsistency index (*I* ^2^) (Zamora et al. 2006). Based on the *I* ^2^ values, heterogeneity was classified as high (> 75%), moderate (50%–75%), or low (< 25%) (Higgins et al. 2003). Finally, we conducted subgroup analyses according to the following characteristics: (1) sample size; (2) administration modes (interviewer‐administered and self‐administered); (3) number of food items; (4) study quality (very good, good, acceptable, and poor); (5) geographical areas.

All statistical analyses were performed using SPSS 30.0 for Windows (IBM Corporation, New York, NY, USA).

## 3 Results

A total of 5247 papers were identified for this study. After removing 1405 duplicate papers, 3842 papers were screened for inclusion based on their title and abstract (Figure 1). After 3665 papers were excluded, the 177 papers that remained underwent full‐text assessment. A total of 134 papers were excluded (Figure 1), with 43 studies included in the meta‐analysis (Babic et al. 2014; Barrat et al. 2012; Block et al. 2006; Boucher et al. 2006; Brunner et al. 2001; Buscemi et al. 2015; Cantin et al. 2016; Dehghan et al. 2012; Deschamps et al. 2009; Fallaize et al. 2014; Garcia et al. 2024; Hebden et al. 2013; Hemiö et al. 2014; Hollis et al. 2017; Jaceldo‐Siegl et al. 2010; Johansson et al. 2002; Kesse‐Guyot et al. 2010; Kowalkowska and Wadolowska 2022; Kristal et al. 2014; Kumanyika et al. 2003; Labonte et al. 2012; Liu et al. 2013; Marventano et al. 2016; Nepal and Hillman 2025; Paalanen et al. 2006; Roddam et al. 2005; Sabir et al. 2022; Sam et al. 2014, 2020; Serban et al. 2021; Shatylo and Solovyova 2024; Sluik et al. 2016; Szmidt et al. 2025; Talegawkar et al. 2015; Turconi et al. 2010; van Dongen et al. 2011; van Dongen et al. 2019; Verbeke et al. 2023; Verger et al. 2017; Visser et al. 2020; Wennberg et al. 2024; Yuan et al. 2017; Zanini et al. 2020).

**FIGURE 1:**
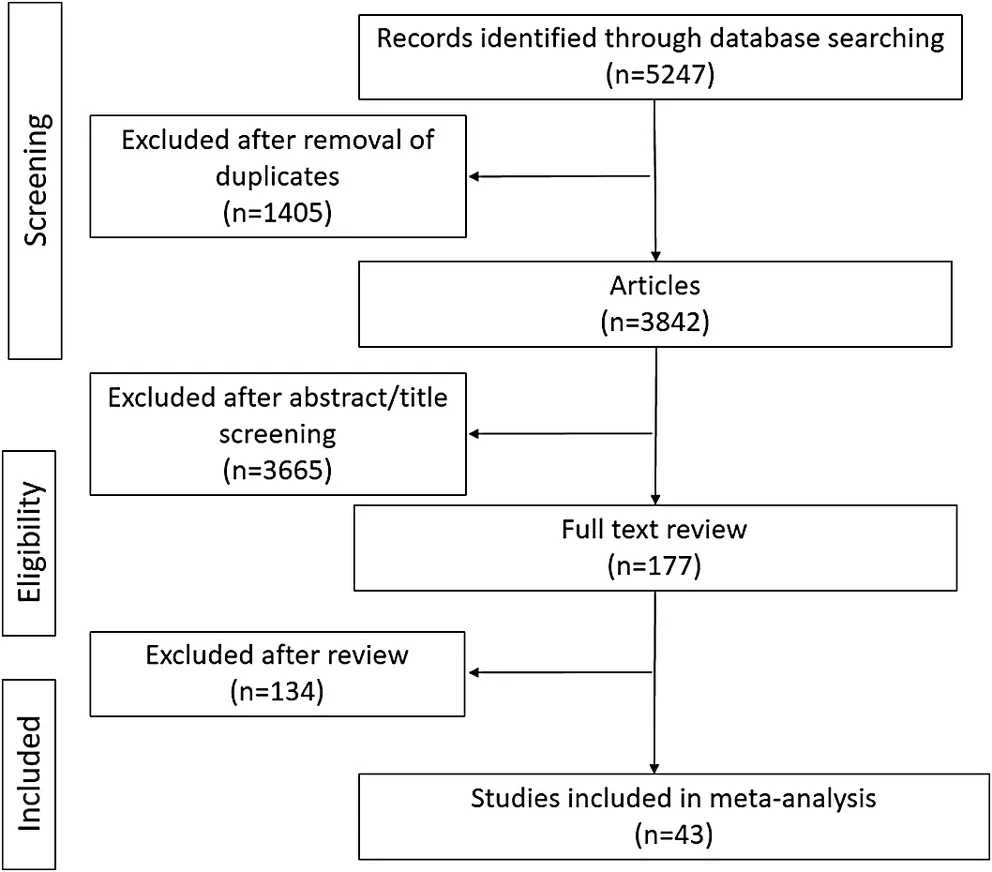
PRISMA flow‐chart research strategy.

The main characteristics of the included studies are reported in Table 1. The overall sample size was 8467 subjects, with a median of 96 participants per study (range 50–632). The study population had a median age of 46.9 years, with a male prevalence of 40%. In 58% of the studies, the 24HR was used as the reference method (Table 1). The median of the FFQ items validated in the studies was 128 items, with considerable variability between studies (range 16–322). The majority of questionnaires were self‐administered, and the most frequently used reference period was less than 12 months (Table 1). Eighty‐six percent of the FFQs employed were semi‐quantitative. From a methodological perspective, study quality was rated as “Good” or “Very Good” in 84% of cases (Table 1). Geographically, most studies were conducted in Europe (67%), followed by North America (26%). The country‐level distribution of included studies within each region is reported in Table S2.

**TABLE 1:** Characteristics of included studies in healthy adults.

Characteristics	Overall	24HRs	FRs
Number of studies	43	25	18
Sample size (median; range)	8467 (96;50–632)	4382 (89;50–550)	4085 (100;50–632)
Men (%)^a^	40 (0–49)	41.7 (0–51)	35 (5–47)
Age^b^ (years; range)	46.9 (21.4–74.7)	53.9 (23.5–71.9)	45.1 (21.4–74.4)
Items of FFQ (median; range)	128 (16–322)	134 (68–322)	127 (16–169)
Administration mode of FFQ^c^			
Interview‐administered	6 (14)	5 (20)	1 (6)
Self‐administered	37 (86)	20 (80)	17 (94)
Reference period^c^			
Previous 12 months	14 (32)	5 (20)	9 (50)
< Previous 12 months	26 (60)	18 (72)	8 (44)
Not available	3 (8)	2 (8)	1 (6)
Type of FFQ^c^			
Semi‐quantitative	37 (86)	22 (88)	15 (83)
Quantitative	6 (14)	3 (12)	3 (17)
Quality of studies^c^			
Very good	12 (28)	8 (28)	4 (27)
Good	24 (56)	19 (68)	5 (33)
Acceptable	5 (11)		5 (33)
Poor	2 (5)	1 (4)	1 (7)
Regions^c^			
Europe	29 (67)	16 (64)	13 (72)
North America	11 (26)	6 (24)	5 (28)
Australia/New Zealand	3 (7)	3 (12)	0 (0)

Abbreviations: 24HRs, 24‐h recalls; FFQ, food frequency questionnaire; FRs, food records.

^a^ Values are median (interquartile range).

^b^ In *n* = 43 studies.

^c^ Values are numbers (%).

As shown in Tables 2 and 3, the correlation coefficients between FFQs and 24HRs varied according to the nutrient assessed, reflecting differences in the predictive performance of the FFQs across nutrients. For macronutrients, the crude correlations for energy, protein, and lipids ranged from 0.413 to 0.454, while the highest values were observed for carbohydrates and fiber (Table 2). Among micronutrients, good performance was observed for vitamin C, calcium, and zinc, whereas iodine and vitamin A were less strongly correlated (Table 2). Heterogeneity among studies was generally high, with *I* ^2^ values often exceeding 75%, indicating substantial variability between the included studies (Table 2). Energy adjustment and correction for intra‐individual error (de‐attenuation) resulted in some changes in the correlation coefficients (Table 3). Overall, energy adjustment produced results similar to those of the crude correlations, whereas de‐attenuation often reduced the strength of the correlations, suggesting that part of the association observed in the crude data might have been influenced by measurement error (Table 3).

**TABLE 2:** Pooled effect estimates (95% CI) and heterogeneity of the correlation coefficients between FFQs and 24‐h recalls for energy and nutrients among healthy adults.

Nutrients	Correlation coefficient (95% CI)	*N*	*I* ^2^
Energy	0.454 (0.352–0.555)	31	91
Carbohydrate	0.519 (0.437–0.601)	31	85
Protein	0.413 (0.345–0.481)	31	77
Lipids	0.437 (0.363–0.510)	30	80
Fiber	0.593 (0.434–0.752)	26	95
SFA	0.454 (0.385–0.522)	27	75
MUFA	0.345 (0.292–0.397)	21	43
PUFA	0.344 (0.291–0.398)	22	48
Vitamin A	0.309 (0.217–0.401)	4	0
Retinol	0.406 (0.237–0.576)	11	87
Vitamin C	0.441 (0.360–0.522)	22	80
Vitamin D	0.433 (0.318–0.547)	12	86
Vitamin E	0.358 (0.260–0.455)	10	80
Vitamin K	—	—	—
Thiamine	0.400 (0.278–0.522)	11	83
Riboflavin	0.568 (0.452–0.685)	8	77
Niacin	0.454 (0.318–0.591)	7	79
Pantothenic Acid	0.638 (0.466–0.810)	2	87
Vitamin B6	0.479 (0.323–0.635)	8	88
Folate	0.433 (0.337–0.529)	15	82
Vitamin B12	0.455 (0.309–0.602)	7	76
Selenium	0.354 (0.183–0.525)	1	—
Magnesium	0.544 (0.448–0.641)	8	68
Calcium	0.445 (0.354–0.536)	23	85
Iron	0.394 (0.299–0.489)	17	82
Iodine	0.103 (−0.037–0.243)	2	0
Zinc	0.524 (0.405–0.643)	8	73
Copper	0.394 (0.229–0.559)	4	78
Potassium	0.436 (0.324–0.549)	10	78
Phosphorus	0.409 (0.319–0.498)	11	60
Sodium	0.382 (0.229–0.535)	11	87
Manganese	—	—	—

*Note:* Data are reported as correlation coefficient and 95% CI.

Abbreviations: CI, confidence interval; *I* ^2^, inconsistency index; MUFA, monounsaturated fatty acids; *N*, number of studies; PUFA, polyunsaturated fatty acids; SFA, saturated fatty acids.

**TABLE 3:** Pooled effect estimates (95% CI) and heterogeneity of the correlation coefficients between FFQs and energy‐adjusted and deattenuated 24‐h recalls among healthy adults.

Nutrients	Energy‐adjusted			De‐attenuated		
	Correlation coefficient (95% CI)	*N*	*I* ^2^	Correlation coefficient (95% CI)	*N*	*I* ^2^
Energy	—	—	—	0.352 (0.198–0.507)	5	65
Carbohydrate	0.525 (0.435–0.614)	21	83	0.440 (0.333–0.547)	12	76
Protein	0.404 (0.314–0.494)	21	83	0.326 (0.252–0.400)	15	69
Lipids	0.452 (0.359–0.544)	21	84	0.367 (0.280–0.454)	15	78
Fiber	0.617 (0.530–0.705)	20	81	0.528 (0.435–0.620)	10	56
SFA	0.444 (0.356–0.531)	17	77	0.382 (0.288–0.475)	7	39
MUFA	0.348 (0.284–0.411)	13	43	0.235 (0.168–0.301)	7	0
PUFA	0.323 (0.245–0.401)	14	67	0.278 (0.212–0.345)	7	0
Vitamin A	0.260 (0.134–0.385)	3	39	0.407 (0.184–0.630)	9	92
Retinol	0.518 (0.215–0.821)	5	91	0.592 (0.333–0.852)	4	86
Vitamin C	0.562 (0.468–0.656)	13	81	0.550 (0.332–0.767)	11	94
Vitamin D	0.456 (0.317–0.594)	9	88	0.358 (0.260–0.455)	7	50
Vitamin E	0.327 (0.210–0.444)	6	67	0.326 (0.168–0.484)	5	81
Vitamin K	—	—	—	—	—	—
Thiamine	0.469 (0.299–0.638)	4	89	0.282 (0.094–0.470)	2	23
Riboflavin	0.578 (0.423–0.733)	4	87	0.650 (0.497–0.803)	2	0
Niacin	0.488 (0.353–0.623)	4	82	0.580 (0.382–0.778)	2	40
Pantothenic Acid	0.638 (0.466–0.810)	2	87	—	—	—
Vitamin B6	0.582 (0.457–0.707)	4	79	0.499 (0.270–0.627)	4	75
Folate	0.465 (0.366–0.564)	5	73	0.455 (0.356–0.555)	7	49
Vitamin B12	0.324 (0.234–0.413)	3	0	0.677 (0.221–1.133)	5	97
Selenium	—	—	—	0.410 (0.207–0.614)	1	—
Magnesium	0.504 (0.397–0.612)	3	74	0.480 (0.401–0.558)	4	5
Calcium	0.536 (0.431–0.641)	16	86	0.435 (0.370–0.499)	13	37
Iron	0.430 (0.294–0.566)	11	90	0.377 (0.325–0.430)	9	0
Iodine	0.151 (−0.094–0.396)	1	—	—	—	—
Zinc	0.530 (0.329–0.732)	5	93	0.401 (0.273–0.529)	3	6
Copper	0.410 (0.190–0.630)	2	92	—	—	—
Potassium	0.370 (0.205–0.536)	3	89	0.331 (0.176–0.486)	4	68
Phosphorus	0.326 (0.138–0.513)	4	91	0.307 (0.203–0.411)	4	0
Sodium	0.172 (0.049–0.296)	5	80	0.340 (0.059–0.621)	4	86
Manganese	—	—	—	—	—	—

*Note:* Data are reported as correlation coefficient and 95% CI.

Abbreviations: CI, confidence interval; *I* ^2^, inconsistency index; MUFA, monounsaturated fatty acids; *N*, number of studies; PUFA, polyunsaturated fatty acids; SFA, saturated fatty acids.

When the reference method consisted of FRs, the crude pooled correlations were generally similar to, or slightly higher than, those observed with 24HRs (Table 4). Heterogeneity among studies was high across most nutrients (Table 4). Similarly to 24HRs, energy adjustment and de‐attenuation for FRs yielded variable shifts in correlation strength depending on the nutrient (Table 5). To assess potential systematic bias in nutrient intake estimates derived from FFQs compared with reference methods, SMDs were calculated. Overall, when FFQs were compared with 24HRs (Table 6), they tended to overestimate the intake of several nutrients. In contrast, comparisons with FRs diaries (Table 7) generally showed smaller differences, indicating closer agreement between FFQs and this reference method. The agreement between correlation coefficients and SMDs for energy and nutrients is shown in Figure 2 (24HRs) and Figure 3 (FRs), respectively for macro‐ and micronutrients. Pooled correlation coefficients between FFQs and 24HRs were greater than 0.30, while SMDs were below 0.50 for energy, lipids, carbohydrates, sodium, calcium, iron, and the vitamins B12, C, D, folate, thiamine, riboflavin, and niacin (Figure 2A,B).

**TABLE 4:** Pooled effect estimates (95% CI) and heterogeneity of the correlation coefficients between FFQs and food records for energy and nutrients among healthy adults.

Nutrients	Correlation coefficient (95% CI)	*N*	*I* ^2^
Energy	0.501 (0.391–0.612)	22	89
Carbohydrate	0.521 (0.438–0.605)	24	83
Protein	0.468 (0.396–0.540)	24	76
Lipids	0.483 (0.395–0.572)	24	84
Fiber	0.598 (0.527–0.669)	23	75
SFA	0.524 (0.445–0.603)	23	79
MUFA	0.434 (0.355–0.513)	22	79
PUFA	0.417 (0.332–0.502)	22	82
Vitamin A	0.433 (0.269–0.597)	11	90
Retinol	0.282 (0.178–0.386)	6	52
Vitamin C	0.479 (0.421–0.538)	22	62
Vitamin D	0.425 (0.293–0.558)	18	90
Vitamin E	0.453 (0.341–0.565)	17	89
Vitamin K	0.416 (0.188–0.645)	4	83
Thiamine	0.500 (0.388–0.612)	15	84
Riboflavin	0.633 (0.532–0.735)	15	80
Niacin	0.468 (0.356–0.580)	10	76
Pantothenic Acid	0.693 (0.494–0.892)	1	—
Vitamin B6	0.482 (0.396–0.569)	14	68
Folate	0.512 (0.449–0.575)	19	64
Vitamin B12	0.530 (0.385–0.676)	15	90
Selenium	0.508 (0.394–0.622)	6	42
Magnesium	0.573 (0.472–0.675)	15	85
Calcium	0.505 (0.428–0.581)	24	78
Iron	0.460 (0.394–0.527)	21	69
Iodine	0.223 (−0.282–0.727)	2	94
Zinc	0.450 (0.353–0.548)	10	68
Copper	0.501 (0.329–0.673)	5	82
Potassium	0.417 (0.310–0.524)	14	84
Phosphorus	0.504 (0.367–0.640)	11	83
Sodium	0.405 (0.308–0.502)	13	71
Manganese	0.464 (0.147–0.780)	2	85

*Note:* Data are reported as correlation coefficient and 95% CI.

Abbreviations: CI, confidence interval; *I* ^2^, inconsistency index; MUFA, monounsaturated fatty acids; *N*, number of studies; PUFA, polyunsaturated fatty acids; SFA, saturated fatty acids.

**TABLE 5:** Pooled effect estimates (95% CI) and heterogeneity of the correlation coefficients between FFQs and energy‐adjusted and deattenuated food records among healthy adults.

Nutrients	Energy‐adjusted			De‐attenuated		
	Correlation coefficient (95% CI)	*N*	*I* ^2^	Correlation coefficient (95% CI)	*N*	*I* ^2^
Energy	—	—	—	0.349 (0.050–0.648)	3	91
Carbohydrate	0.559 (0.456–0.611)	14	88	0.463 (0.291–0.635)	4	87
Protein	0.434 (0.357–0.512)	14	78	0.391 (0.177–0.605)	4	92
Lipids	0.467 (0.406–0.528)	14	64	0.495 (0.310–0.680)	4	89
Fiber	0.634 (0.572–0.696)	13	60	0.500 (0.411–0.590)	3	22
SFA	0.531 (0.454–0.607)	12	76	0.522 (0.329–0.715)	2	90
MUFA	0.432 (0.347–0.516)	11	79	0.395 (0.266–0.523)	2	78
PUFA	0.356 (0.275–0.436)	12	79	0.434 (0.102–0.765)	2	97
Vitamin A	0.340 (0.286–0.394)	8	0	0.348 (0.187–0.509)	4	85
Retinol	0.280 (0.165–0.394)	5	73	0.458 (0.380–0.536)	1	—
Vitamin C	0.489 (0.424–0.554)	14	69	0.433 (0.369–0.497)	4	18
Vitamin D	0.395 (0.304–0.486)	10	75	0.681 (0.459–0.902)	4	92
Vitamin E	0.357 (0.288–0.425)	14	72	0.397 (0.228–0.566)	4	87
Vitamin K	0.416 (0.360–0.473)	3	0	0.441 (0.300–0.582)	2	82
Thiamine	0.386 (0.320–0.452)	10	45	0.316 (−0.101–0.733)	2	89
Riboflavin	0.632 (0.547–0.717)	10	67	0.617 (−0.037–1.271)	2	95
Niacin	0.359 (0.250–0.468)	6	72	—	—	—
Pantothenic Acid	0.633 (0.434–0.832)	1	—	—	—	—
Vitamin B6	0.414 (0.295–0.533)	8	84	0.372 (0.300–0.443)	4	31
Folate	0.516 (0.482–0.550)	14	0	0.395 (0.246–0.543)	4	83
Vitamin B12	0.377 (0.301–0.454)	8	60	0.515 (0.190–0.841)	4	97
Selenium	0.431 (0.350–0.511)	4	18	0.317 (0.227–0.408)	1	—
Magnesium	0.616 (0.543–0.690)	13	74	0.576 (0.461–0.691)	4	71
Calcium	0.527 (0.450–0.604)	14	78	0.544 (0.490–0.598)	4	0
Iron	0.476 (0.397–0.555)	13	77	0.438 (0.202–0.673)	4	93
Iodine	0.618 (0.501–0.736)	2	0	—	—	—
Zinc	0.371 (0.301–0.441)	8	48	0.374 (0.165–0.584)	4	92
Copper	0.389 (0.236–0.542)	3	72	0.354 (0.295–0.413)	2	0
Potassium	0.517 (0.426–0.607)	9	80	0.422 (0.331–0.513)	1	—
Phosphorus	0.562 (0.503–0.621)	5	0	0.518 (0.320–0.717)	4	91
Sodium	0.287 (0.169–0.405)	7	81	0.193 (0.102–0.284)	1	—
Manganese	0.642 (0.567–0.716)	3	4	0.570 (0.479–0.661)	1	—

*Note:* Data are reported as correlation coefficient and 95% CI.

Abbreviations: CI, confidence interval; *I* ^2^, inconsistency index; MUFA, monounsaturated fatty acids; *N*, number of studies; PUFA, polyunsaturated fatty acids; SFA, saturated fatty acids.

**TABLE 6:** Pooled effect estimates (95% CI) and heterogeneity of standardized mean differences between FFQs and 24‐h recalls among healthy adults.

Nutrients	SMD (95% CI)	*N*	*I* ^2^	*p*
Energy	−0.273 (−1.117–0.571)	24	100	0.52
Carbohydrate	0.022 (−0.400–0.445)	24	98	0.91
Protein	0.552 (0.309–0.796)	24	95	< 0.001
Lipids	0.381 (0.167–0.594)	24	94	< 0.001
Fiber	0.828 (0.355–1.300)	21	99	< 0.001
SFA	0.497 (0.132–0.862)	22	98	0.008
MUFA	0.665 (0.199–1.13)	19	98	0.005
PUFA	0.814 (0.353–1.27)	18	98	< 0.001
Vitamin A	0.523 (0.069–0.977)	5	93	0.024
Vitamin C	0.456 (0.197–0.71)	15	95	< 0.001
Vitamin D	0.169 (−0.148–0.487)	8	95	0.29
Vitamin E	1.24 (0.307–2.179)	7	99	0.009
Thiamine	0.364 (0.172–0.555)	9	84	< 0.001
Riboflavin	0.314 (0.068–0.560)	7	90	0.012
Niacin	0.247 (0.175–0.319)	6	0	< 0.001
Vitamin B6	0.582 (0.063–1.10)	7	98	0.028
Folate	0.036 (−0.951–1.022)	12	100	0.94
Vitamin B12	0.062 (−0.210–0.333)	4	79	0.56
Magnesium	0.742 (0.303–1.181)	7	97	< 0.001
Calcium	−0.077 (−0.833–0.739)	17	100	0.85
Iron	0.064 (−0.305–0.433)	11	97	0.73
Potassium	1.038 (0.567–1.509)	7	97	< 0.001
Sodium	0.382 (0.130–0.635)	9	91	0.003

*Note:* Data are reported as SMDs and 95% CI.

Abbreviations: CI, confidence interval; *I* ^2^, inconsistency index; MUFA, monounsaturated fatty acids; *N*, number of studies; PUFA, polyunsaturated fatty acids; SFA, saturated fatty acids; SMD, standardized mean difference.

**TABLE 7:** Pooled effect estimates (95% CI) and heterogeneity of standardized mean differences between FFQs and food records among healthy adults.

Nutrients	SMD (95% CI)	*N*	*I* ^2^	*p*
Energy	0.319 (−0.045–0.682)	12	97	0.08
Carbohydrate	0.362 (−0.087–0.811)	12	98	0.11
Protein	0.283 (0.004–0.563)	12	95	0.047
Lipids	0.190 (−0.110–0.489)	12	96	0.21
Fiber	0.434 (−0.09–0.96)	11	99	0.10
SFA	0.150 (−0.117–0.417)	12	95	0.27
MUFA	0.147 (−0.159–0.452)	12	96	0.34
PUFA	0.207 (−0.183–0.598)	12	98	0.29
Vitamin A	0.163 (−0.057–0.383)	7	84	0.14
Vitamin C	0.307 (−0.039–0.654)	11	97	0.08
Vitamin D	0.269 (−0.09–0.627)	6	92	0.14
Vitamin E	0.060 (−0.222–0.343)	9	95	0.67
Thiamine	0.338 (−0.120–0.797)	8	96	0.14
Riboflavin	0.298 (−0.016–0.612)	8	92	0.06
Niacin	0.376 (−0.025–0.778)	7	95	0.06
Vitamin B6	0.415 (0.196–0.633)	8	84	< 0.001
Folate	0.329 (−0.044–0.702)	11	97	0.08
Vitamin B12	0.105 (−0.011–0.222)	7	42	0.76
Magnesium	0.027 (−0.292–0.347)	8	96	0.86
Calcium	0.092 (−0.312–0.496)	12	98	0.65
Iron	1.218 (0.382–2.054)	12	99	0.004
Potassium	0.168 (−0.691–1.027)	10	99	0.70
Sodium	0.089 (−0.638–0.815)	9	99	0.81

*Note:* Data are reported as SMDs and 95% CI.

Abbreviations: CI, confidence interval; *I* ^2^, inconsistency index; MUFA, monounsaturated fatty acids; *N*, number of studies; PUFA, polyunsaturated fatty acids; SFA, saturated fatty acids; SMD, standardized mean difference.

**FIGURE 2:**
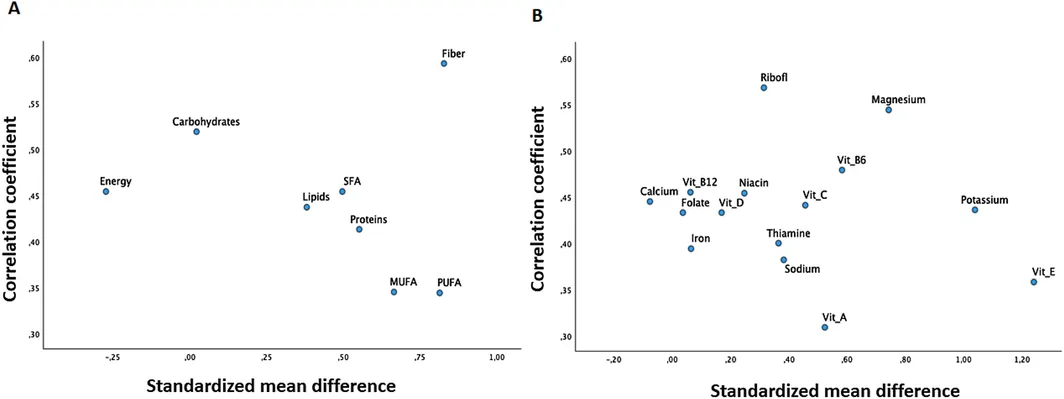
The agreement of the pooled correlation coefficients and standardized mean differences when 24‐h recalls were used as reference methods. (A) For energy and macronutrients; (B) for micronutrient. Overall, pooled correlation coefficients were > 0.30 and standardized mean differences < 0.50, indicating acceptable agreement between food frequency questionnaires and 24‐h recalls. In panel A, this pattern was observed for energy, lipids, and carbohydrates, while in panel B similar results were found for sodium, calcium, iron, and vitamins B12, C, D, folate, thiamine, riboflavin, and niacin. MUFA, monounsaturated fatty acids; PUFA, polyunsaturated fatty acids; Ribofl, riboflavin; SFA, saturated fatty acids; Vit_A, vitamin A; Vit_B12, vitamin B12; Vit_B6, vitamin B6; Vit_C, vitamin C; Vit_D, vitamin D; Vit_E, vitamin E.

**FIGURE 3:**
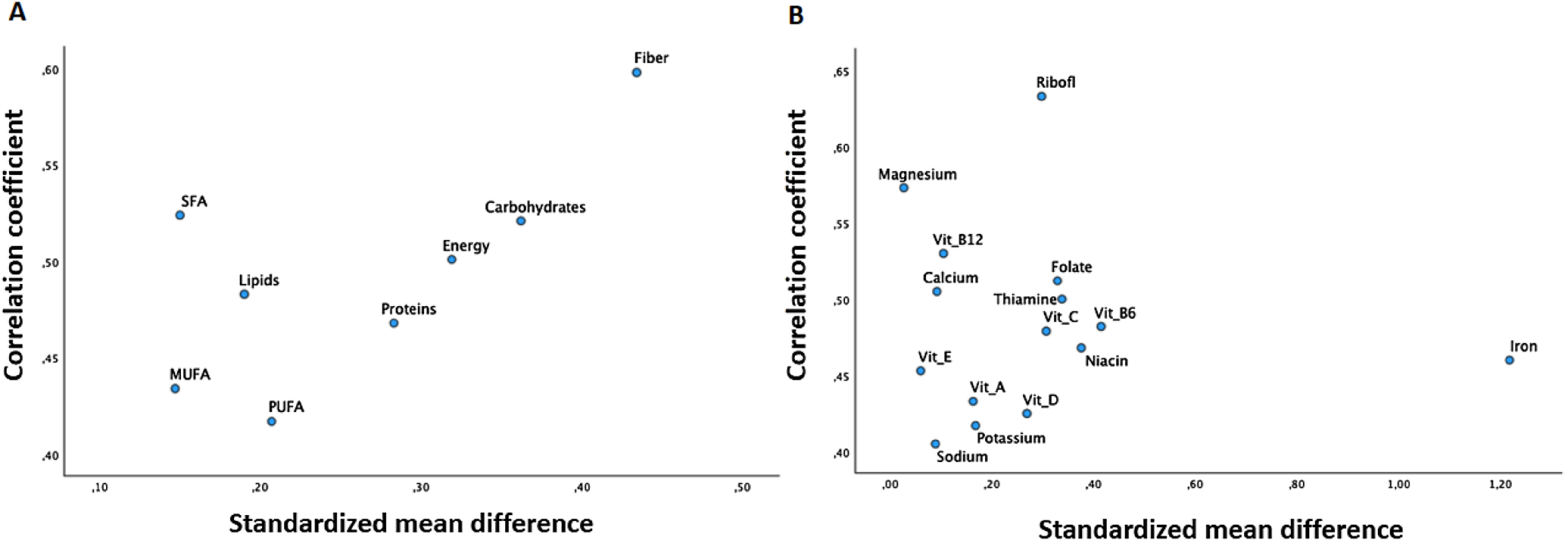
The agreement of the pooled correlation coefficients and standardized mean differences when food records were used as reference methods. (A) For energy and macronutrients; (B) for micronutrient. Overall, pooled correlation coefficients exceeded 0.30 and standardized mean differences remained below 0.50, indicating acceptable agreement between food frequency questionnaires and food records. In panel A, this pattern was observed for energy and all macronutrients, while in panel B similar results were found for most micronutrients, with the exception of iron, which showed lower agreement. MUFA, monounsaturated fatty acids; PUFA, polyunsaturated fatty acids; Ribofl, riboflavin; SFA, saturated fatty acids; Vit_A, vitamin A; Vit_B12, vitamin B12; Vit_B6, vitamin B6; Vit_C, vitamin C; Vit_D, vitamin D; Vit_E, vitamin E.

For FRs, pooled correlation coefficients between FFQs and the reference method exceeded 0.30, while SMDs remained below 0.50 for energy, all macronutrients, and most micronutrients, with the exception of iron (Figure 3A,B).

The subgroup analysis based on sample size did not reveal substantial differences in the pooled correlation coefficients between studies with sample sizes below or above the median (Table S3). In both groups, the correlations between the FFQ and the reference method were generally moderate for the main macronutrients. However, studies with larger sample sizes showed significantly higher heterogeneity values, with *I* ^2^ often exceeding 80%–90%, suggesting greater methodological variability among larger studies (Table S3). In contrast, studies with sample sizes below the median presented lower levels of heterogeneity (Table S3).

The subgroup analysis based on the mode of questionnaire administration shows that interview‐administered FFQs tend to present higher pooled correlation coefficients for several nutrients compared with self‐administered FFQs, particularly for energy, carbohydrates, proteins, and lipids (Table S4). However, for some nutrients such as fiber and saturated fatty acids, self‐administered FFQs show comparable or slightly higher correlations (Table S4). In addition, heterogeneity among studies remains high in both groups, with *I* ^2^ values frequently exceeding 70%–90%, indicating considerable methodological variability among the included studies (Table S4).

The subgroup analysis based on the number of questionnaire items shows that FFQs with a number of items above the median tend to present slightly higher pooled correlations for several nutrients compared with questionnaires with fewer items (Table S5).

The subgroup analysis based on the methodological quality of the studies shows that studies classified as having very good quality tend to report slightly higher pooled correlation coefficients compared with other studies (Table S6). This pattern is observable for most nutrients, including energy, carbohydrates, proteins, lipids, and fiber.

Finally, the subgroup analysis based on the geographical area of the studies highlights some differences in the relative validity of FFQs between studies conducted in Europe and those carried out in other regions of the world (Table S7).

## 4 Discussion

The present systematic review and meta‐analysis provide a comprehensive assessment of the relative validity of FFQs and identify methodological characteristics associated with their performance in assessing habitual dietary intake. Rather than questioning the established role of FFQs in nutritional epidemiology, our findings help clarify the conditions under which these instruments may provide more informative estimates and the dietary assessment purposes for which they are best suited. In this context, the transition from paper‐based to digital formats reflects the growing demand for more scalable and standardized approaches to dietary assessment in nutritional research (Illner et al. 2012).

The decision to include studies conducted exclusively in Europe, North America, Australia, and New Zealand was driven by the need to ensure homogeneity in reference dietary patterns. In addition, studies including fewer than 50 participants were excluded to reduce the influence of small‐sample studies, as a sample size of at least 50 participants, and preferably larger (around 100 or more), is generally recommended for dietary validation studies (Cade et al. 2002; Willett 2013).

The systematic comparison conducted in the meta‐analysis (Table 1) shows that FFQs have been primarily validated against 24HRs. The pooled correlation coefficients reported in Tables 2 and 4 indicate moderate‐to‐good validity for most nutrients. For total energy, correlation coefficients were 0.454 (vs. 24HRs) and 0.501 (vs. FRs). These values are consistent with the international literature, which suggests that FFQs tend to capture the qualitative composition of the diet more effectively than the absolute quantitative accuracy of energy intake (Willett 2013). It is noteworthy that correlations are generally higher when the reference method is the FRs (Table 4); this phenomenon can be explained by the prospective nature of the diary, which reduces memory‐related biases common to both FFQs and 24HRs.

The results indicate that the FFQ is particularly effective in ranking intake of carbohydrates and dietary fiber. The high correlation observed for fiber is of particular clinical relevance, given its protective role in non‐communicable chronic diseases (Aea et al. 2019).

The tool demonstrated robust performance in capturing SFA intake, whereas slightly lower correlations were observed for MUFA and PUFA. This likely reflects the inherent difficulty participants have in accurately identifying types of added fats and condiments, a well‐known limitation of self‐reported dietary assessment methods (Cade et al. 2002).

The present findings should be considered in relation to Cui et al. (2023) to our knowledge the largest existing meta‐analysis of FFQ validity in adults (130 studies, 21,494 participants; PubMed and Web of Science only, January 2000–April 2020; global geographic scope spanning Asia, Europe, North America, South America, Oceania, and Africa). The present study is methodologically related to this prior work—both pool Fisher's *z*‐transformed correlation coefficients and standardized mean differences by reference method type, and both apply the Serra‐Majem quality‐scoring framework (Serra‐Majem et al. 2009)—and extends it in three respects: (i) it restricts the geographic scope to Western countries with comparable dietary assessment infrastructure, in contrast to the globally pooled estimates of Cui et al. (2023), yielding effect estimates more directly interpretable for Western/European epidemiological research contexts; (ii) it extends the literature search through February 2026, capturing FFQ validation studies published in the 6 years following the Cui et al. (2023) search cut‐off, including a growing number of digital/web‐based FFQs; and (iii) it incorporates Scopus as a third bibliographic database in addition to PubMed and Web of Science.

The analysis of micronutrients revealed considerable variability. Some nutrients showed excellent correlations, such as magnesium (correlation coefficients = 0.573 vs. FRs) and riboflavin (correlation coefficients = 0.633). In contrast, elements such as sodium exhibited weaker correlations (correlation coefficients = 0.382 vs. 24HRs), likely due to high day‐to‐day variability and discretionary salt use, which are difficult to quantify using a food frequency questionnaire (Table 2).

An important implication concerns the intended application of FFQs assessing overall dietary intake. As nutrient‐specific FFQs were excluded, our findings primarily reflect comprehensive instruments for habitual diet assessment. FFQ performance was relatively consistent for energy and macronutrients, particularly carbohydrates, but more variable for micronutrients, with stronger correlations for magnesium and riboflavin and weaker performance for sodium and iodine. Thus, overall‐diet FFQs appear suitable for characterizing dietary patterns and ranking energy and macronutrient intake, whereas specific micronutrient estimates should be interpreted cautiously and may require targeted validation or complementary assessment methods.

Analysis of SMDs also suggested the presence of systematic bias in nutrient intake estimates obtained via FFQ (Tables 6 and 7). In particular, the FFQ tended to overestimate the intake of certain nutrients compared with reference methods, especially when compared with 24HRs. This phenomenon has been widely described in the literature and can be attributed to several factors, including participants' difficulty in estimating average portion sizes and the tendency to overreport foods perceived as healthy (Freedman et al. 2011). However, this issue can be mitigated statistically (Kipnis et al. 2003).

Data from our meta‐analysis (Table 1) confirm that 86% of modern studies use self‐administered FFQs, indicating excellent participant acceptability.

In addition, we conducted subgroup analyses based on sample size, FFQ administration mode, number of items, study quality, and the regions in which the studies were conducted. The subgroup analysis based on sample size did not reveal substantial differences in pooled correlation coefficients between studies with sample sizes below or above the median. Overall, these results indicate that sample size does not appear to substantially affect the relative validity of FFQs, although it may contribute to variability among the studies included in the meta‐analysis. The subgroup analysis based on FFQ administration mode showed that interviewer‐administered FFQs tended to yield higher pooled correlation coefficients for several nutrients compared with self‐administered FFQs, particularly for energy, carbohydrates, proteins, and lipids. This finding suggests that the presence of an interviewer may improve the quality of the collected data, likely by reducing errors in question comprehension and enhancing portion size estimation. In addition, the subgroup analysis based on the number of items in the questionnaire indicated that FFQs with a number of items above the median tended to show slightly higher pooled correlations for several nutrients compared with questionnaires with fewer items. This finding is consistent with the FFQ methodological literature, which suggests that including a greater number of foods in the questionnaire better captures habitual dietary variability, thereby improving the tool's ability to rank individuals according to nutrient intake. Regarding the subgroup analysis based on study methodological quality, the meta‐analysis results suggest that higher methodological quality in validation studies may contribute to improved agreement between FFQs and reference methods, likely due to more rigorous procedures in dietary data collection, management of measurement errors, and statistical analysis. Finally, for several nutrients, studies conducted in Europe showed slightly higher pooled correlation coefficients compared with studies conducted in other geographic regions.

Taken together, these subgroup findings provide practical guidance for FFQ development and validation. Interviewer administration may reduce comprehension and portion‐size estimation errors, while broader food‐list coverage may improve performance, although at the cost of greater respondent burden. The stronger correlations observed in higher‐quality studies highlight the importance of rigorous reference methods, standardized data collection, and measurement‐error management. In contrast, sample size had little influence once an adequate validation sample was reached. Future studies should therefore focus on the conditions under which FFQs provide the most informative estimates for their intended epidemiological application.

## 5 Study Strengths and Limitations

The present meta‐analysis has several strengths. It is based on a PRISMA‐compliant systematic search across three bibliographic databases (PubMed, Web of Science, Scopus) extended through February 2026. It jointly evaluates relative validity (correlation coefficients) and systematic bias (standardized mean differences) against two distinct reference methods (24HRs and FRs), across an extensive panel of macro‐ and micro‐nutrients. It applies a standardized methodological quality assessment (Serra‐Majem et al. 2009) and includes five pre‐specified subgroup analyses exploring candidate sources of heterogeneity (sample size, administration mode, number of items, study quality, geographic area).

An intrinsic limitation of the present meta‐analysis concerns the geographical selection of the included studies. The analysis focused exclusively on research conducted primarily in Europe and North America, excluding contexts such as Africa, Asia, and South America. Although this choice limits the global generalizability of the results, it was intended to preserve the consistency of the dietary pattern under investigation.

Including populations with substantially different dietary assessment contexts, food composition databases, and validation procedures could have introduced additional sources of heterogeneity, making the interpretation of pooled estimates more challenging.

Despite the robustness of the data, the high heterogeneity observed (*I* ^2^ > 80% for many nutrients in Tables 2 and 4) should also be considered. Very high heterogeneity (*I* ^2^ frequently > 75%–90%) is a well‐documented feature of the FFQ validation literature and is not unique to the present study. For example, the largest meta‐analysis of FFQ validity to date (Cui et al. 2023; 130 studies, 21,494 participants, global geographic scope) reported validity coefficients ranging as widely as 0.220–0.770 (vs. 24HRs) and 0.173–0.735 (vs. FRs). Notably, restricting the geographic scope of the present meta‐analysis to a more culturally and methodologically homogeneous set of Western countries did not eliminate high heterogeneity, suggesting that instrument‐level factors (FFQ length, administration mode, reference period, nutrient‐specific measurement error) are likely a more dominant driver of heterogeneity in this literature than population or cultural diversity alone. Consistent with standard meta‐analytic practice under substantial heterogeneity, we used random‐effects pooling, which estimates a distribution of true effects rather than a single fixed parameter, and pre‐specified subgroup analyses to explore candidate sources of variability; we consider this the appropriate approach for this literature rather than a reason to forgo quantitative synthesis. This variability may be attributed to differences in the studied populations (median age 46.9 years, with very wide ranges), the number of items included in the FFQs (ranging from 16 to 322), and the different reference periods used (Table 1). Furthermore, systematic under‐reporting remains an intrinsic limitation of self‐reported dietary assessment methods, which digitalization alone cannot completely eliminate (Freedman et al. 2011). An additional limitation is that many included studies assessed FFQ validity mainly through correlation coefficients. Although these measures are useful for evaluating relative validity and the ability to rank individuals according to dietary intake, they do not directly assess absolute agreement between FFQ estimates and reference methods. Restricting eligibility to studies reporting Pearson or Spearman correlation coefficients was a deliberate methodological choice necessary to enable quantitative pooling: this was the validity metric most consistently and completely reported across the FFQ validation literature, whereas complementary measures of absolute agreement (e.g., Bland–Altman analysis, classification into quintiles/tertiles) are reported using highly heterogeneous formats across studies, precluding meaningful quantitative synthesis. Standardized mean differences (Tables 6 and 7), also computed in the present study, provide a complementary indication of systematic bias in absolute intake levels. Accordingly, our findings should be interpreted as pertaining to the relative validity of FFQs (i.e., their ability to rank individuals) rather than to their absolute agreement with reference methods.

## 6 Conclusion

In conclusion, this meta‐analysis supports the relative validity of FFQs as tools for assessing habitual dietary intake, particularly for classifying individuals based on their habitual intake, which is one of their main applications in nutritional epidemiology. Among overall dietary FFQs, performance was relatively consistent for energy and macronutrients, while greater variability was observed for micronutrients, indicating that estimates of specific micronutrient intakes should be interpreted with greater caution. Subgroup analyses also suggest that questionnaire characteristics and validation procedures influence FFQ performance: interviewer administration, greater food coverage, and higher methodological quality tended to be associated with stronger correlations with reference methods. These findings provide practical guidance for future FFQ development and validation and highlight the importance of selecting and designing FFQs based on the specific dietary exposure of interest. Further studies should determine whether digital formats and other methodological innovations can improve validity while maintaining the efficiency, scalability, and feasibility that make FFQs particularly valuable in large‐scale epidemiological research. FQs in terms of validity and overall performance.

## Author Contributions

**Carmelo Pujia:** conceptualization, investigation, methodology, data curation, writing – original draft. **Yvelise Ferro:** investigation, formal analysis, writing – original draft, methodology. **Alessia Placanica:** investigation, data curation. **Rossella Bruno:** investigation, data curation. **Fiorella Guadagni:** supervision, validation, writing – review and editing. **Giulia Malaguarnera:** supervision, validation, writing – review and editing. **Elisa Mazza:** investigation, methodology. **Tiziana Montalcini:** formal analysis, writing – original draft, supervision, validation, project administration. **Arturo Pujia:** conceptualization, formal analysis, writing – original draft, writing – review and editing, supervision, validation, project administration.

## Funding

The authors have nothing to report.

## Disclosure

*Declaration of generative AI and AI‐assisted technologies in the writing process*: The authors used ChatGPT 4.0 to improve the language and readability of the manuscript. Post‐processing, the authors carefully vetted the generated output and take total responsibility for the accuracy and originality of the content.

## Conflicts of Interest

The authors declare no conflicts of interest.

## Supporting information


**Table S1.** The search strategy used for each database.
**Table S2.** Country‐level distribution of included studies within each geographic region.
**Table S3.** Subgroup analysis—pooled effect estimates (95% CI) and heterogeneity of correlation coefficients between FFQs and reference methods (24‐h recalls and food records) according to sample size.
**Table S4.** Subgroup analysis—pooled effect estimates (95% CI) and heterogeneity of correlation coefficients between FFQs and reference methods (24‐h recalls and food records) according to administration mode of FFQs.
**Table S5.** Subgroup analysis—pooled effect estimates (95% CI) and heterogeneity of correlation coefficients between FFQs and reference methods (24‐h recalls and food records) according to the number of items of FFQs.
**Table S6.** Subgroup analysis—pooled effect estimates (95% CI) and heterogeneity of correlation coefficients between FFQs and reference methods (24‐h recalls and food records) according to the quality of studies.
**Table S7.** Subgroup analysis—pooled effect estimates (95% CI) and heterogeneity of correlation coefficients between FFQs and reference methods (24‐h recalls and food records) according to the geographical areas.

## Data Availability

The datasets in the present study can be obtained from the corresponding author upon a reasonable request.
