# Supplementary material for: Relative Validity of Food Frequency Questionnaires in Adult Populations: A Systematic Review and Meta‐Analysis
Source: Food Sci Nutr. 2026 Sep 30;14(10):e72381. doi: 10.1002/fsn3.72381 (PMC13624932; doi:10.1002/fsn3.72381)
Supplement: Supplementary file 1 [file FSN3-14-e72381-s001.docx]

**Supplementary Materials**

| **Supplemental Table 1.** *The search strategy used for each database* | | |
| --- | --- | --- |
| **Database** | **Search string** | **Filters** |
| PubMed | (FFQ[Title/Abstract] OR Food frequency questionnaire[Title/Abstract]) AND (Validation[Title/Abstract] OR validity[Title/Abstract]) | 2000/01/01–2026/02/01; English language |
| Web of Science | FFQ or Food frequency questionnaire AND validation or validity | Timespan 2000-01-01 to 2026-02-01 (Index Date); English language |
| Scopus | TITLE-ABS-KEY(ffq OR food frequency questionnaire) AND TITLE-ABS-KEY(validation OR validity) | PUBYEAR > 1999 AND PUBYEAR < 2027; English language |

| **Supplemental Table 2.** *Country-level distribution of included studies within each geographic region* | | | |
| --- | --- | --- | --- |
| **Region**  **(n)** | **Country** | **Studies**  **(n)** | **Studies (first author, year)** |
| Europe (29) | France | 4 | Barrat 2012; Deschamps 2009; Kesse-Guyot 2010; Verger 2017 |
|  | United Kingdom | 4 | Brunner 2001; Fallaize 2014; Hollis 2017; Roddam 2005 |
|  | Italy | 4 | Buscemi 2015; Marventano 2016; Turconi 2010; Zanini 2020 |
|  | Netherlands | 4 | Sluik 2016; van Dongen 2011; van Dongen 2019; Visser 2020 |
|  | Poland | 3 | Dehghan 2012; Kowalkowska 2022; Szmidt 2025 |
|  | Finland | 2 | Hemiö 2014; Paalanen 2006 |
|  | Sweden | 2 | Johansson 2002; Wennberg 2024 |
|  | Croatia | 1 | Babić 2014 |
|  | Serbia | 1 | Garcia 2024 |
|  | Norway | 1 | Sabir 2022 |
|  | Romania | 1 | Serban 2021 |
|  | Ukraine | 1 | Shatylo 2024 |
|  | Belgium | 1 | Verbeke 2023 |
| North America (11) | United States | 7 | Block 2006; Jaceldo-Siegl 2010; Kristal 2014; Kumanyika 2003; Nepal 2025; Talegawkar 2015; Yuan 2017 |
|  | Canada | 4 | Boucher 2006; Cantin 2016; Labonté 2012; Liu 2013 |
| Australia/New Zealand (3) | Australia | 1 | Hebden 2013 |
|  | New Zealand | 2 | Sam 2012; Sam 2020 |
| N, number. | | | |

| **Supplemental Table 3.** *Subgroup analysis - Pooled effect estimates (95% CI) and heterogeneity of correlation coefficients between FFQs and reference methods (24-hour recalls and food records) according to sample size* | | | | | |
| --- | --- | --- | --- | --- | --- |
| **Nutrients** | **Sample size** | **Correlation coefficients** | **CI 95%** | **N** | **I²** |
| Energy | ≤ median | 0.418 | 0.38 – 0.45 | 30 | 49.9 |
| Energy | > median | 0.394 | 0.37 – 0.42 | 23 | 95.5 |
| Carbohydrate | ≤ median | 0.474 | 0.44 – 0.51 | 29 | 28.8 |
| Carbohydrate | > median | 0.464 | 0.44 – 0.48 | 25 | 92.3 |
| Protein | ≤ median | 0.401 | 0.36 – 0.44 | 29 | 26.8 |
| Protein | > median | 0.375 | 0.35 – 0.40 | 25 | 88.1 |
| Lipids | ≤ median | 0.413 | 0.38 – 0.45 | 29 | 56.0 |
| Lipids | > median | 0.421 | 0.40 – 0.44 | 25 | 90.6 |
| Fiber | ≤ median | 0.513 | 0.48 – 0.55 | 25 | 68.4 |
| Fiber | > median | 0.578 | 0.56 – 0.60 | 23 | 96.1 |
| SFA | ≤ median | 0.442 | 0.41 – 0.48 | 28 | 48.6 |
| SFA | > median | 0.472 | 0.45 – 0.49 | 22 | 88.7 |
| MUFA | ≤ median | 0.368 | 0.33 – 0.41 | 23 | 49.9 |
| MUFA | > median | 0.371 | 0.35 – 0.39 | 20 | 83.1 |
| PUFA | ≤ median | 0.346 | 0.31 – 0.39 | 23 | 38.9 |
| PUFA | > median | 0.375 | 0.35 – 0.40 | 21 | 84.5 |
| CI, confidence interval; N, number of studies; I^2^, inconsistency index; SFA, saturated fatty acids; MUFA, monounsaturated fatty acids; PUFA, polyunsaturated fatty acids. | | | | | |

| **Supplemental Table 4.** *Subgroup analysis* **-** *Pooled effect estimates (95% CI) and heterogeneity of correlation coefficients between FFQs and reference methods (24-hour recalls and food records) according to administration mode of* *FFQs* | | | | | |
| --- | --- | --- | --- | --- | --- |
| **Nutrients** | **Administration mode** | **Correlation coefficients** | **CI 95%** | **N** | **I²** |
| Energy | Self-administered | 0.379 | 0.36 – 0.40 | 45 | 87.5 |
| Energy | Interview-administered | 0.599 | 0.55 – 0.65 | 8 | 95.0 |
| Carbohydrate | Self-administered | 0.457 | 0.44 – 0.48 | 46 | 84.8 |
| Carbohydrate | Interview-administered | 0.557 | 0.51 – 0.61 | 8 | 83.3 |
| Protein | Self-administered | 0.375 | 0.36 – 0.40 | 46 | 76.9 |
| Protein | Interview-administered | 0.444 | 0.38 – 0.50 | 8 | 83.4 |
| Lipids | Self-administered | 0.409 | 0.39 – 0.43 | 46 | 79.7 |
| Lipids | Interview-administered | 0.519 | 0.47 – 0.57 | 8 | 91.7 |
| Fiber | Self-administered | 0.575 | 0.56 – 0.59 | 43 | 93.0 |
| Fiber | Interview-administered | 0.361 | 0.28 – 0.44 | 5 | 70.6 |
| SFA | Self-administered | 0.472 | 0.45 – 0.49 | 43 | 80.5 |
| SFA | Interview-administered | 0.371 | 0.30 – 0.44 | 7 | 64.1 |
| MUFA | Self-administered | 0.370 | 0.35 – 0.39 | 37 | 74.7 |
| MUFA | Interview-administered | 0.373 | 0.30 – 0.45 | 6 | 64.3 |
| PUFA | Self-administered | 0.372 | 0.35 – 0.39 | 38 | 76.4 |
| PUFA | Interview-administered | 0.320 | 0.24 – 0.40 | 6 | 36.8 |
| CI, confidence interval; N, number of studies; I^2^, inconsistency index; SFA, saturated fatty acids; MUFA, monounsaturated fatty acids; PUFA, polyunsaturated fatty acids. | | | | | |

| **Supplemental Table 5.** *Subgroup analysis* **-** *Pooled effect estimates (95% CI) and heterogeneity of correlation coefficients between FFQs and reference methods (24-hour recalls and food records) according to the number of items of FFQs* | | | | | |
| --- | --- | --- | --- | --- | --- |
| **Nutrients** | **Number of items** | **Correlation coefficients** | **CI 95%** | **N** | **I²** |
| Energy | ≤ median | 0.387 | 0.36 – 0.41 | 28 | 82.9 |
| Energy | > median | 0.413 | 0.39 – 0.44 | 25 | 93.8 |
| Carbohydrate | ≤ median | 0.434 | 0.41 – 0.46 | 27 | 79.9 |
| Carbohydrate | > median | 0.492 | 0.47 – 0.51 | 27 | 87.6 |
| Protein | ≤ median | 0.364 | 0.34 – 0.39 | 27 | 65.6 |
| Protein | > median | 0.396 | 0.37 – 0.42 | 27 | 84.0 |
| Lipids | ≤ median | 0.393 | 0.37 – 0.42 | 27 | 72.4 |
| Lipids | > median | 0.440 | 0.42 – 0.46 | 27 | 88.1 |
| Fiber | ≤ median | 0.465 | 0.44 – 0.49 | 26 | 83.0 |
| Fiber | > median | 0.644 | 0.62 – 0.66 | 22 | 94.5 |
| SFA | ≤ median | 0.458 | 0.43 – 0.48 | 26 | 62.0 |
| SFA | > median | 0.470 | 0.45 – 0.49 | 24 | 86.7 |
| MUFA | ≤ median | 0.371 | 0.34 – 0.40 | 19 | 76.7 |
| MUFA | > mediana | 0.370 | 0.35 – 0.40 | 24 | 71.0 |
| PUFA | ≤ median | 0.347 | 0.32 – 0.38 | 20 | 69.8 |
| PUFA | > median | 0.383 | 0.36 – 0.41 | 24 | 77.1 |
| CI, confidence interval; N, number of studies; I^2^, inconsistency index; SFA, saturated fatty acids; MUFA, monounsaturated fatty acids; PUFA, polyunsaturated fatty acids. | | | | | |

| **Supplemental Table 6.** *Subgroup analysis* - *Pooled effect estimates (95% CI) and heterogeneity of correlation coefficients between FFQs and reference methods (24-hour recalls and food records) according to the quality of studies* | | | | | |
| --- | --- | --- | --- | --- | --- |
| **Nutrients** | **Quality studies** | **Correlation coefficients** | **CI 95%** | **N** | **I²** |
| Energy | Very good | 0.512 | 0.46 – 0.56 | 12 | 82 |
| Energy | Other studies | 0.421 | 0.39 – 0.45 | 31 | 92 |
| Carbohydrate | Very good | 0.553 | 0.50 – 0.61 | 12 | 74 |
| Carbohydrate | Other studies | 0.498 | 0.46 – 0.53 | 31 | 88 |
| Protein | Very good | 0.452 | 0.40 – 0.51 | 12 | 70 |
| Protein | Other studies | 0.396 | 0.36 – 0.43 | 31 | 84 |
| Lipids | Very good | 0.471 | 0.42 – 0.52 | 12 | 78 |
| Lipids | Other studies | 0.432 | 0.40 – 0.46 | 30 | 86 |
| Fiber | Very good | 0.628 | 0.57 – 0.69 | 11 | 83 |
| Fiber | Other studies | 0.571 | 0.53 – 0.61 | 32 | 94 |
| SFA | Very good | 0.503 | 0.45 – 0.56 | 11 | 72 |
| SFA | Other studies | 0.442 | 0.41 – 0.48 | 32 | 86 |
| MUFA | Very good | 0.382 | 0.33 – 0.43 | 9 | 60 |
| MUFA | Other studies | 0.351 | 0.32 – 0.38 | 28 | 79 |
| PUFA | Very good | 0.401 | 0.35 – 0.45 | 9 | 64 |
| PUFA | Other studies | 0.347 | 0.31 – 0.38 | 29 | 80 |
| CI, confidence interval; N, number of studies; I^2^, inconsistency index; SFA, saturated fatty acids; MUFA, monounsaturated fatty acids; PUFA, polyunsaturated fatty acids. | | | | | |

| **Supplemental Table 7.** *Subgroup analysis - Pooled effect estimates (95% CI) and heterogeneity of correlation coefficients between FFQs and reference methods (24-hour recalls and food records) according to the geographical areas* | | | | | |
| --- | --- | --- | --- | --- | --- |
| **Nutrients** | **Regions** | **Correlation coefficients** | **CI 95%** | **N** | **I²** |
| Energy | Europe | 0.443 | 0.42 – 0.47 | 36 | 89.2 |
| Energy | Other countries | 0.335 | 0.30 – 0.37 | 17 | 91.6 |
| Carbohydrate | Europe | 0.479 | 0.46 – 0.50 | 38 | 75.6 |
| Carbohydrate | Other countries | 0.445 | 0.42 – 0.47 | 16 | 92.3 |
| Protein | Europe | 0.401 | 0.38 – 0.42 | 37 | 70.5 |
| Protein | Other countries | 0.352 | 0.32 – 0.38 | 17 | 85.8 |
| Lipids | Europe | 0.404 | 0.38 – 0.43 | 37 | 81.0 |
| Lipids | Other countries | 0.441 | 0.41 – 0.47 | 17 | 87.1 |
| Fiber | Europe | 0.580 | 0.56 – 0.60 | 32 | 93.4 |
| Fiber | Other countries | 0.541 | 0.52 – 0.57 | 16 | 91.2 |
| SFA | Europe | 0.438 | 0.41 – 0.46 | 33 | 65.6 |
| SFA | Other countries | 0.500 | 0.47 – 0.53 | 17 | 88.2 |
| MUFA | Europe | 0.353 | 0.32 – 0.38 | 30 | 71.3 |
| MUFA | Other countries | 0.398 | 0.36 – 0.43 | 13 | 76.6 |
| PUFA | Europe | 0.327 | 0.30 – 0.35 | 31 | 64.7 |
| PUFA | Other countries | 0.435 | 0.40 – 0.47 | 13 | 78.6 |
| CI, confidence interval; N, number of studies; I^2^, inconsistency index; SFA, saturated fatty acids; MUFA, monounsaturated fatty acids; PUFA, polyunsaturated fatty acids. | | | | | |
